# Reexamining the Mycovirome of *Botrytis* spp.

**DOI:** 10.3390/v16101640

**Published:** 2024-10-21

**Authors:** Hugo Muñoz-Suárez, Ana Ruiz-Padilla, Livia Donaire, Ernesto Pérez Benito, María A. Ayllón

**Affiliations:** 1 Centro de Biotecnología y Genómica de Plantas, Universidad Politécnica de Madrid—Instituto Nacional de Investigación y Tecnología Agraria y Alimentaria (UPM-INIA/CSIC), Pozuelo de Alarcón, 28223 Madrid, Spain; hugo.munoz.suarez@alumnos.upm.es (H.M.-S.); ana.ruizp@upm.es (A.R.-P.); 2 Department of Stress Biology and Plant Pathology, Centro de Edafología y Biología Aplicada del Segura (CEBAS)-CSIC, 30100 Murcia, Spain; ldonaire@cebas.csic.es; 3 Instituto de Investigación en Agrobiotecnología (CIALE), Departamento de Microbiología y Genética, Universidad de Salamanca, C/Río Duero, 12, Villamayor, 37185 Salamanca, Spain; epbenito@usal.es; 4 Departamento de Biotecnología-Biología Vegetal, Escuela Técnica Superior de Ingeniería Agronómica, Alimentaria y de Biosistemas, Universidad Politécnica de Madrid (UPM), 28040 Madrid, Spain

**Keywords:** mycoviruses, mycovirome, *Narnaviridae*, *Botrytis cinerea*, *Botrytis prunorum*, RNA silencing, antiviral mechanism, bioinformatics, high throughput sequencing

## Abstract

*Botrytis* species cause gray mold disease in more than 200 crops worldwide. To control this disease, chemical fungicides are usually applied. However, more sustainable control alternatives should be explored, such as the use of hypovirulent mycovirus-infected fungal strains. To determine the mycovirome of two *Botrytis* species, *B. cinerea* and *B. prunorum*, we reanalyzed RNA-Seq and small RNA-Seq data using different assembly programs and an updated viral database, aiming to identify new mycoviruses that were previously not described in the same dataset. New mycoviruses were identified, including those previously reported to infect or be associated with *B. cinerea* and *Plasmopara viticola*, such as Botrytis cinerea alpha-like virus 1 and Plasmopara viticola lesion-associated ourmia-like virus 80. Additionally, two novel narnaviruses, not previously identified infecting *Botrytis* species, have been characterized, tentatively named Botrytis cinerea narnavirus 1 and Botrytis narnavirus 1. The analysis of small RNAs suggested that all identified mycoviruses were targeted by the antiviral fungal mechanism, regardless of the viral genome type. In conclusion, the enlarged list of newly found viruses and the application of different bioinformatics approaches have enabled the identification of novel mycoviruses not previously described in *Botrytis* species, expanding the already extensive list.

## 1. Introduction

Fungi of the genus *Botrytis* (phylum *Ascomycota*, family *Sclerotiniaceae*) are necrotrophic plant pathogens, which preferentially develop and infect damaged or senescent tissues, resulting in a progressive decay, and eventually causing their death [1]. *Botrytis cinerea* Pers. Fr. (teleomorph *Botryotinia fuckeliana* (de Bary) Whetzel) is the only generalist species in the genus, capable of infecting over 1000 plant species, including more than 200 crops worldwide; in contrast, *B. prunorum* primarily infects *Prunus* spp. [1]. *B. cinerea* causes gray mold in important agronomical and ornamental crops, leading to significant economic losses in field plants and post-harvest products [2,3]. The pathogen produces gray mycelium that sporulates abundantly on infected tissue [4].

*B. cinerea* is mainly controlled by the application of chemical fungicides; however, its genome plasticity results in the emergence of resistant strains [5,6,7] that, together with the growing public concern about crop residues, have made it necessary to explore alternatives to chemical control. In the search for sustainable control methods, biological control agents have been researched over the last few years [8]. In this field, the discovery of mycoviruses, or fungal viruses, and their potential applications in biological control strategies against plant pathogenic fungi have stimulated theirstudy in *B. cinerea*.

Since the first report of a virus-caused disease in fungi was documented in 1962 [9], a large number of mycoviruses have been described that infect the major fungal taxa [10,11]. To date, most of the mycoviruses described have been found to have double-stranded RNA (dsRNA) or positive single-stranded RNA (ssRNA(+)) genomes [12,13,14]. Additionally, mycoviruses with single-stranded DNA (ssDNA) [15] and negative single-stranded RNA (ssRNA(−)) genomes have also been described [12,13,14,16]. The symptoms caused by mycoviruses infecting plant pathogenic fungi are very varied [17,18,19,20]. Most are latent and harmless to their hosts, although others can cause hypovirulence, reducing the virulence of the fungus in the plant host [21,22]. Cryphonectria hypovirus 1 is the first case described of a mycovirus successfully controlling its fungal host *Cryphonectria parasitica* [23,24,25] and it is still used as a biocontrol agent in chestnut trees.

Mycoviruses of *B. cinerea* have been extensively studied over the years. First, dsRNA purification led to the discovery of ssRNA(+)/(−) and dsRNAs viruses [26,27,28,29,30]. Then, the development of next-generation sequencing techniques allowed scientists to analyze fungal transcriptomes and characterize sequences of the first ssRNA(−) mycovirus infecting *B. cinerea* [31]. In addition, novel ssRNA(+) viruses infecting *B. cinerea* such as mitoviruses [32], ourmia-like viruses [33] and narnaviruses [34], among others [35], have been identified. Several HTS (High Throughput Sequencing)-based techniques have been used for virus identification, including total RNA, small RNAs (sRNAs), and dsRNA sequencing. Total RNA sequencing (RNA-Seq) or transcriptome sequencing have mainly been performed by using the Illumina platform, which retrieves reads of approximately 150 bp, and detect viruses with ssRNA, dsRNA, and ssDNA genomes [31,34,36,37,38]. Small RNA sequencing (sRNA-Seq) has mainly been performed using 454 Life Science technology and has resulted in reads of approximately 20–26 nucleotides [32]. In addition, the sequencing of fragmented initiator-linked dsRNA (FLDS) has led to the determination of RNA viral sequences [39]. Furthermore, the analysis of HTS data requires sophisticated bioinformatic pipelines to process and interpret the vast amounts of sequencing data. Numerous bioinformatic tools and pipelines have been developed for this purpose, each tailored to different aspects of mycovirus research [40,41]. In addition, specialized software packages like VirFind and VirusDetect [42,43] have been designed specifically for viral sequence detection and classification within HTS datasets.

In a previous work, the mycoviromes of three field isolates of two species of the fungus *Botrytis* were analyzed [32]. By sequencing total RNA and sRNA, a total of fifteen mycoviruses, of ten different species, were identified. In this work, we have reexamined the RNA-Seq and sRNA-Seq data derived from V446, V448, and Pi258.8 isolates in an attempt to expand the mycoviromes of these three *Botrytis* field isolates. For this purpose, an improved bioinformatics pipeline for mycovirus detection, previously implemented by Ruiz-Padilla et al. [34], and a updated viral database were used.

## 2. Materials and Methods

### 2.1. Fungal Isolates and Culture Conditions

The *Botrytis* field isolates used in this project were *B. cinerea* Pi258.8 and V448, and *B. prunorum* V446. Isolate Pi258.8 was obtained from pepper from a greenhouse in Almería [29,32], whereas isolates V446 and V448 were obtained from grapes from a vineyard in Roa (Burgos) [31,33,44]. These isolates were stored in 20% glycerol at −80 °C. Stock cultures were maintained on PDA (potato dextrose agar) plates at 4 °C. Subsequently, fresh mycelium was obtained by placing agar pieces with mycelium from the stock culture in 100 mL of PDB (potato dextrose broth) and incubating the mixture in the dark at 23 °C for 10 days.

### 2.2. RNA-Seq and sRNA-Seq Data

The raw data from cDNA libraries of total RNA and sRNA obtained by Donaire et al. [31,32,33] were used. Previously obtained data were saved in the Sequence Read Archive (SRA) at NCBI: BioProject PRJNA325479, Biosamples SAMN05233215 (Pi258.8), SAMN05233216 (V446), SAMN05233218 (V448), SRA SRX1838896 (Pi258.8), SRX1838897 (V446) and SRX1838898 (V448).

### 2.3. RNAseq Data Analysis

The RNA-Seq data files for each of the three *Botrytis* field isolates were used for the in silico search for mycoviruses in the samples, as were previously used [31,32,33]. The analysis of these RNA-Seq libraries was performed by adapting the bioinformatics pipeline described in [34]. Briefly, this pipeline is divided into four main steps: (1) sequence cleaning, (2) de novo assembly of the cleaned sequences, (3) viral sequence identification and (4) mapping of the cleaned reads to the identified viral sequences. Sequence cleaning was performed using Bbtools software (Version 38.42) to remove TrueSeq adapters, low-quality sequences (Q30) and ribosomal sequences. These reads were de novo assembled using *Trinity* (Version 2.11.0) [45] and *Spades* (Version 3.15.2) [46] and reassembled using *CAP3* [47]. *Trinity* and *Spades* carry out the assembly from reads. *CAP3* uses assembled contigs and finds common sequences to generate longer ones. Contigs were blasted using local *BLASTx* (Basic Local Alignment Search Tool) [48] (Version 0.9.24) loaded from the *DIAMOND* module [49] (Version 2.14). *BLAST* was performed against a complete and updated local database of proteins of viral origin obtained from GenBank containing all viral sequences until April 2023. A search was carried out using the following parameters: cut-off expected value of 0.00001 and additional sensitivity. The viral database was fragmented into ten parts. The total number of viral sequences were divided into chunks of 1,000,000 using the AWK command. Each contig was used for independent *BLAST* searches, and the top two hits with the highest identities and coverage were selected for further analysis. These selected contigs were blasted against the complete non-redundant (nr) protein NCBI database to confirm that they were viral sequences. In order to analyze the distribution of reads mapping with viral genomes, clean reads were mapped against the viral sequences found using *BWA* (Version 0.7.17) [50], *Samtools* (Version 1.9) [51] and the *Geneious* map tool (Version 2013) [52]. Mappings were visualized with *Geneious* and the *IGV* (Integrative Genomics Viewer) (Version 2.16) [53].

### 2.4. sRNA-Seq Data Analysis

The sRNA-Seq libraries for each of the three field isolates of *Botrytis* were also re-aligned. Reads between 20 and 26 nucleotides in length were filtered and subsequently mapped against the mycoviral genomes, previously detected by RNA-Seq, using *Geneious*, allowing only one mismatch with the reference genome. The sRNAs were used to determine whether these viruses were being targeted by the fungal gene-silencing machinery. They also served to validate the presence of mycoviruses identified with the RNA-Seq data and, in some cases, to complete their genomic sequence by using *Geneious* alignments. The number of viral sRNAs (vsRNAs) was normalized to the Reads Per Kilobase of contigs per Million mapped reads (RPKM) value, which was calculated using next formula: Total of mapped reads × 10^9^/(total reads × contig length (kb). The Fragments Per Kilobase of contigs per Million mapped reads (FPKM) value was calculated for paired-end reads from RNA-Seq data with the same formula.

### 2.5. Three-Dimensional Structure and Putative Function of Selected Mycoviral Proteins

The 3D structures of the ORF2-encoded proteins of Botrytis narnavirus 1 (BNV1) and Botrytis cinerea narnavirus 1 (BcNV1) were predicted with AlphaFold2 [54]. The quality of the predicted structures was analyzed by QMEAN (https://swissmodel.expasy.org/qmean/) (accessed on 1 July 2024) [55]. To find structural homology, the predicted 3D structures were compared to database proteins using Phyre2 [56] and DALI (Distance matrix alignment) [57].

### 2.6. In Vivo Detection of Selected Viruses

RNA extraction was performed using the method described in [29], with some modifications. Fresh mycelium was dried using pressure and sterile filter paper and total RNA was purified from one gram of dried mycelium using TRIZOL reagent (Invitrogen) [29,32]. To verify the presence of novel mycoviruses found in strains V446 and V448, the total RNA from each field isolate was used as a template for One-Step Reverse Transcription (RT)–Detection PCR (Takara) as follows: 200 ng of total RNA was mixed with 1× of 2× One-Step Mix, 0.75 U of RT-PCR enzyme mix and 0.1 μM of specific forward and reverse primers. Reactions were incubated following the protocol: 1 cycle at 50 °C for 30 min; 1 cycle at 94 °C 2 min; 30 cycles at 94 °C for 30 s, 55 °C for 30 s and 72 °C for 30 s; and 1 final cycle of elongation at 72 °C for 7 min. Samples were stored at −20 °C. PCR controls were included to discard that the detected sequences were genome-encoded. The reactions were carried out using specific primers for each of the mycoviruses BNV1, BcNV1 and PvaOLV80 (Table 1). Primers to detect BNV1 and BcNV1 were designed based on the sequences of both genomic segments of each virus. Furthermore, primers BNV1 R1 and BcNV1 F2 were used to target a common conservative region of both viruses (Table 1). The strain Pi258.8, free of these mycoviruses (BcNV1, BNV1 and PvaOLV80), was used as a negative control. PCR products were analyzed by electrophoresis on 2% agarose gel stained with Redsafe (iNtRON). Bands containing the amplified products were purified with the NucleoSpin Gel & PCR Clean-up gel kit (Macherey-Nagel kit) and nucleotide sequences were determined by Sanger sequencing.

### 2.7. Phylogenetic Analysis

A maximum likelihood (ML) phylogenetic tree was constructed based on the multiple amino acid sequence alignment MUSCLE [58]. The ML phylogenetic trees were constructed using the IQ-TREE (version 1.6.12) [59] with 1000 replicates of ultrafast bootstrap [60] and the best-fit amino acid substitution model (VT+I+G4), identified using ModelFinder [61]. Viruses classified in the family *Mitoviridae* were used as outgroups.

## 3. Results

### 3.1. Analysis of RNA-Seq Data from Botrytis spp.

This study is a reanalysis of the RNA-Seq data from field isolates of *Botrytis* spp. Pi258.8, V446 and V448 [31,32,33] in which mycoviruses had already been found. For the identification of previously undetected mycoviruses, the protocols of Ruiz-Padilla et al. [34] and Chiapello et al. [62] were adapted. The identification of the mycoviruses previously found in these samples indicated that the bioinformatic pipeline had been followed correctly, validating our results.

Reads were successfully cleaned from adapters and low-quality sequences. In the assembling step, the efficiency of the assembly was tested using two different software platforms, *Trinity* and *Spades*. The results showed different numbers of contigs or sequences assembled with each of them, and this number was reduced after the reassembly with *CAP3* (Table 2). The highest number of contigs was obtained for isolate V448, followed by V446, and finally, Pi258.8, using both assemblers. Additionally, *Trinity* generates around 30% more assembled sequences for all isolates than *Spades*.

Using the assembled contigs, a search for viral sequences was performed by *BLASTx*. Around 25% of the assembled contigs showed identity (*BLAST*x-hits) with viral sequences in the NCBI database (Table 2). The number of reassembled contigs was reduced with respect to the original contigs after applying *CAP3*. For contigs assembled by *Trinity*, this number decreased by 10%, while for *Spades*, the reduction was just 3%. Regarding length ranges, no differences were shown between contigs before and after the *CAP3* treatment (Supplementary Table S1). N50 values were calculated to define the sequence length of the shortest contig at 50% of the total assembly length (Supplementary Table S2). The N50 values were quite similar for both assemblers; however, *Trinity* assemblies showed slightly higher values than *Spades* ones. Reassembly was performed only with sequences showing viral identity, which may explain the low percentage of contigs reduced after reassembly with *CAP3*.

### 3.2. Determination of the Mycovirome of Botrytis spp.

The mycoviromes of the Pi258.8, V446, and V448 isolates were determined from the analysis of RNA-Seq data by comparing all contigs against the NCBI database. Among them, contigs that mapped specifically to mycoviruses were selected and further analyzed (Table 3).

In field isolate Pi258.8, the four mycoviruses already found in the 2017 analysis [32] were also identified in this new analysis, Botrytis cinerea mitovirus 1 (BcMV-1), Botrytis cinerea mitovirus 2 (BcMV-2), Botrytis cinerea mitovirus 3 (BcMV-3), and Grapevine-associated narnavirus 1 (GaNV-1), thereby validating the pipeline used. In addition, Botrytis cinerea alpha-like virus 1 (BcAV1) [34] was also identified in this isolate. In field isolate V446, the previously identified mycoviruses, Botrytis ourmia-like virus (BOLV) [33], Sclerotinia sclerotiorum dsRNA mycovirus-L (SsNsV-L) [32] and the same partial sequence of a endornavirus (LN827950), were found, as well as two other mycoviruses with identity to Plasmopara viticola lesion-associated ourmia-like virus 80 (PavOLV80) [63] and Sclerotinia sclerotiorum narnavirus 3 (SsNV-3) [63]. Finally, in field isolate V448, all previously described mycoviruses were identified [32]: BcMV-1, BcMV-2, BcMV-3, Botrytis cinerea mitovirus 4 (BcMV-4), GaNV-1, Sclerotinia sclerotiorum mitovirus 3 (SsMV-3), Botrytis virus F (BVF) [64], SsNsV-L, and Botrytis cinerea negative-stranded RNA virus 1 (BcNSRV-1) [31]. Additionally, two more mycoviruses were also found with identity to BcAV1 [34] and SsNV-3 [63]. Therefore, the new pipeline and the use of an up-to-date viral database allowed the identification of three already known mycoviruses that were not previously identified in the first analysis. Interestingly, the majority of the mycoviruses found in these samples have a genome of (+)ssRNA, showing the higher abundance of these types of mycoviruses.

The next analysis of the newly identified mycoviruses (BcAV1, PavOLV80, and SsNV-3) in this study, not previously identified in 2017, was focused on two main points: the determination of the complete CDS and a significant part of the genome and the search for conserved domains in the encoded proteins. As a first approach, mycoviral sequences showing identity to BcAV1, PavOLV80 and SsNV-3 found in the three field isolates were compared with their reference genomes (Table 4). Mapping data for these mycoviruses are included in Supplementary Figure S1.

BcAV1 was identified in isolates Pi258.8 and V448. In the case of Pi258.8, the assembled contig of 8045 nts covered the full genome sequence, showing 96% identity at the nucleotide level and 99% at amino acid level with the BcAV1 sequence isolate described by Ruiz-Padilla et al. [34] (Table 4). However, in V448, the contigs obtained were partial but also had a sequence identity at the nucleotide and amino acid level of 95% and 99%, respectively, with respect to the BcALV-1 sequence [34] (Table 4). This confirms that these are two mycoviruses of the same species as BcAV1, sharing more than 95% identity in their nucleotide sequence. The analysis of putative proteins revealed the RdRp domain, as reported previously [34]. The genome schemes of BcAV1–Pi258.8 and BcAV1–V448 are represented in Supplementary Figure S2A.

In isolate V446, a contig with a high nucleotide sequence identity (96%) corresponding to the complete mycovirus genome of PavOLV80 (Genbank accession number PP776574) was found (Table 4). Then, it would be considered a mycovirus of the same species *Deltascleroulivirus betaplasmoparae* within the genus *Deltascleroulivirus* of the family *Botourmiaviridae* (https://ictv.global/report/chapter/botourmiaviridae/botourmiaviridae/deltascleroulivirus) (Accessed on 1 September 2024) [65]. This new mycovirus will be referred to as PvaOLV80 isolate V446 (PvaOLV80-V446). In addition, conserved RdRp motifs were found in the protein encoded by this sequence, as previously reported by Chiapello et al. [62]. The genome scheme of PvaPLV80-V446 is represented in Supplementary Figure S2A. The presence of PavOLV80 in isolate V446 was confirmed by RT-PCR with specific primers, as well as its absence in the fungus genome (Table 1; Supplementary Figure S2B).

Finally, contigs with sequence identity to SsNV-3 were found in both the V446 and V448 isolates. SsNV-3 is a mycovirus with a bisegmented genome, consisting of segment 1 (SsNV-3 RNA1), which encodes an RdRp, and segment 2 (SsNV-3 RNA2), supposedly coding for a capsid protein (CP), as previously suggested [63]. For both V446 and V448, RNA2 was longer than the complete genomic sequence of SsNV-3 RNA2, whereas RNA1 was not complete. Moreover, in no case did the sequence identities exceed 83% at the nucleotide or amino acid level with respect to the reference mycovirus genome (Table 4). In the case of the two RNA genomic sequences of SsNV-3-V446, the sequence identity at the amino acid level was 83% for genomic RNA1 and 61% for genomic RNA2 (Table 4). The mycoviral sequences found in V448 had a sequence identity at the amino acid level of 80% with RNA1 and 58% with RNA2 (Table 4). Furthermore, when comparing the sequences of each segment identified in both isolates, it was observed that the sequence identity was 78% in RNA1 and 68% in RNA2. These results indicate that both mycoviruses might be considered different species from one another, and in turn, different species from SsNV-3 within the same genus *Narnavirus*. These novel mycoviruses were named Botrytis cinerea narnavirus 1 (BcNV1) (isolate V448) (Genbank accession number PP776568, PP776569) and Botrytis narnavirus 1 (BNV1) (isolate V446) (Genbank accession number PP776566, PP776567).

### 3.3. Analysis of Virus-Derived sRNAs

Previously, Donaire and Ayllón [32] showed that (+)ssRNA mycoviruses BcMV-1, BcMV-2, BcMV-3, BcMV-4, SsMV-3, GaNV-1, BOLV, and BVF, dsRNA mycovirus SsNsV-L, and (−)ssRNA mycovirus BcNSRV-1 were targeted by the fungal gene silencing machinery. To demonstrate that the newly identified mycoviruses were also cleaved by the fungus, a new analysis was performed using sRNA sequencing data. For the sRNA-Seq analysis, reads of 20 to 26 nucleotides in length were used. The three isolates had a similar number of sRNAs, being more numerous in Pi258.8 and less in V446. The size of the more abundant sRNAs was variable between the mycoviruses and *Botrytis* species. sRNAs of 21 nts were the most abundant for all mycoviruses, except for SsNsV-L-V446, which contains a slightly higher amount of 22 nt sRNAs (Supplementary Figure S3).

**Figure 1:**
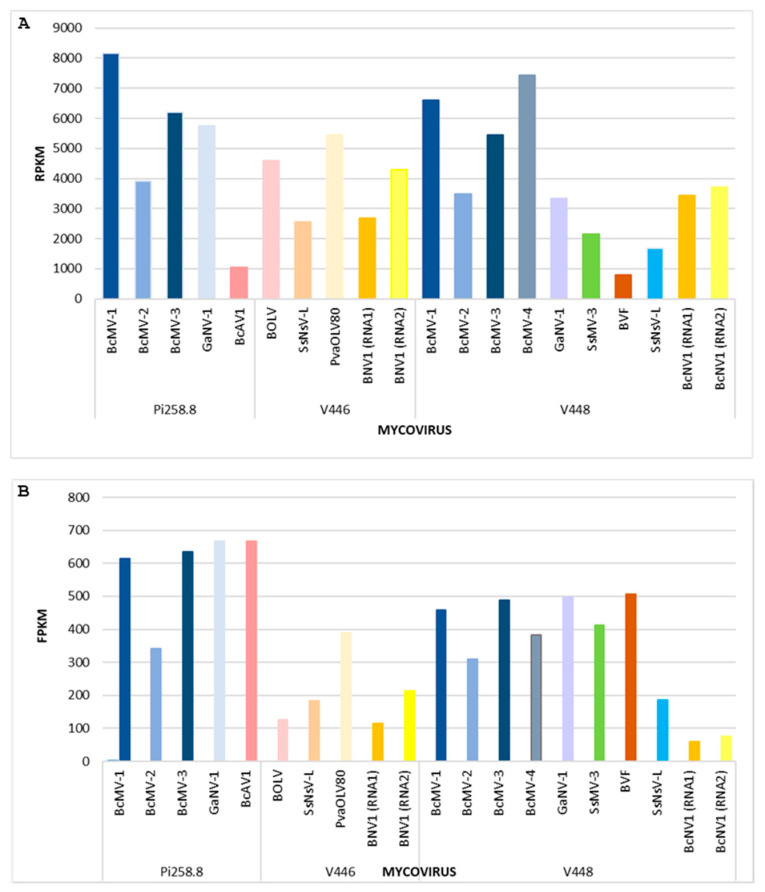
Representation of (**A**) total number of vsRNA reads mapping to each mycovirus measured in RPKM and (**B**) total number of reads of RNA-Seq mapping to each mycovirus measured in FPKM, in the fungal isolates.

The sRNA reads were mapped against the mycoviral genomes identified in each isolate (Table 3). A highly covered map for the full viral sequences was achieved in all the mycoviral sequences, indicating that all mycovirus genomes were targeted by the fungal gene silencing machinery to generate vsRNAs. The vsRNAs were mapped with different frequency along the full viral genomes, meaning that some regions had more hits than others. The vsRNAs sequences were additionally used to complete the genome of the non-completed mycoviruses genomic sequences, as in our previous study [32]. The number of vsRNAs and clean RNA-Seq reads that were mapped to each of the mycoviral genomes were normalized to RPKM and FPKM, respectively (Figure 1).

A comparison of the levels of vsRNAs in each mycovirus (Figure 1A) shows that, in general, a higher number of vsRNAs were derived from mitoviruses. The number of vsRNAs was the highest in BcMV-1 (Pi258.8), followed by BcMV-4 (V448), BcMV-1 (V448), BcMV-3 (Pi258.8), GaNV-1 (Pi258.8), PavOLV80 (V446) and BcMV-3 (V448), and BOLV (446). The number of vsRNAs derived from BcAV1 and BVF was very low, even when the accumulation of both mycoviruses was the highest in their corresponding fungal isolates. The data for the vsRNAs can be related to the accumulation levels of each mycovirus (Figure 1B), based on the normalized number of clean RNA-Seq reads that mapped onto the viral genomes. In general, regarding mitoviruses and botourmiaviruses, a clear relationship can be observed between the accumulation level of the genomic RNA and vsRNAs (Figure 1). However, in BcAV1 (Pi258.8), BOLV (V446), GaNV-1 (V448), SsMV3 (V448) and BVF (V448), this correlation does not occur. In the Pi258.8 isolate, the accumulation of three mitoviruses and BcAV1 was higher than the remaining mycoviruses in the three isolates. In V448, mitoviruses and BVF were also predominant over the rest of the V448 mycoviruses. However, in the V446 isolate, PavOLV80 was the most accumulated mycovirus (Figure 1). The accumulation of the novel mycovirus BNV1 (V446) (based on the total number of RNA-Seq reads) was higher than that of BcNV1 (V448), while the total number of derived vsRNAs was higher for BcNV1 RNA1 and lower for BcNV1 RNA2 (Figure 1). However, the ratio of vsRNAs for both segments corresponded approximately to the ratio of particular segment accumulation (Figure 1).

### 3.4. Molecular Characterization of Novel Mycoviruses

**Figure 2:**
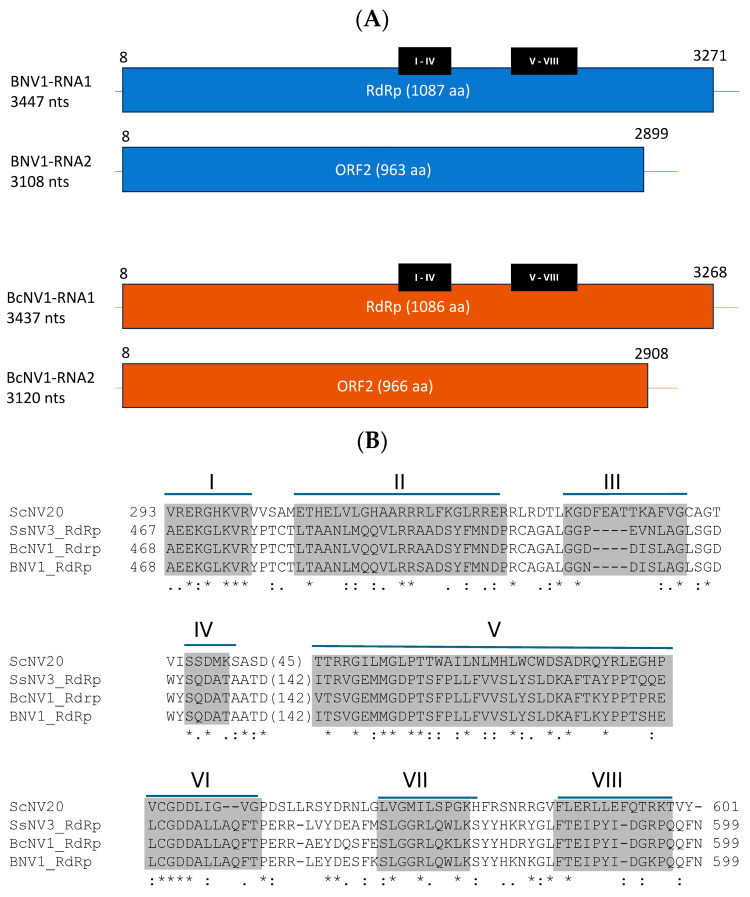
Characterization of novel mycoviruses. (**A**) Scheme of genomes of BNV1 and BcNV1. Positions of conserved motifs are marked in black bars. (**B**) Alignment of conserved motifs of RdRp. (Asterisks (*) show positions with a single fully conserved residue, (.) means: positions with conservation among groups of weakly similar properties. and double points (:) positions with conservation among groups of strongly similar properties).

The novel mycoviruses, BcNV1 and BNV1, identified in this work in the *Botrytis* field isolates V446 and V448, respectively, were further characterized. BNV1 RNA1 is 3447 nt long, with 49% GC content and a single ORF from position 8 to 3271 that encodes for a protein of 1087 aa with a theoretical molecular weight of 121 kDa (Figure 2A). This encoded protein presents conserved domains of the RdRp of (+)ssRNA viruses, including the GDD catalytic motif (Figure 2B) [66]. RNA2 is 3108 nt long, with 49% GC content and with a single ORF extending from nt position 8 to 2899 that encodes for a 963 aa protein, with a molecular weight of 105 kDa, and has an unknown function. Similarly, BcNV1 RNA1 is 3437 nt in length, with 50% GC content and a 3261 nt ORF from position 8 to 3268, which encodes for a 1087 aa RdRp with a molecular weight of 121 kDa. BcNV1 RNA2, is 3120 nt long, with 50% GC content and a 2901 nt ORF, from position 8 to 2908, that encodes for a 966 aa protein with a molecular weight of 106 kDa (Figure 2A). Both mycoviruses have regions in their genomes that show 100% identity between the corresponding segments. These identical regions are up to 62 nucleotides long in RNA1 and 13 nucleotides long in RNA2.

**Figure 3:**
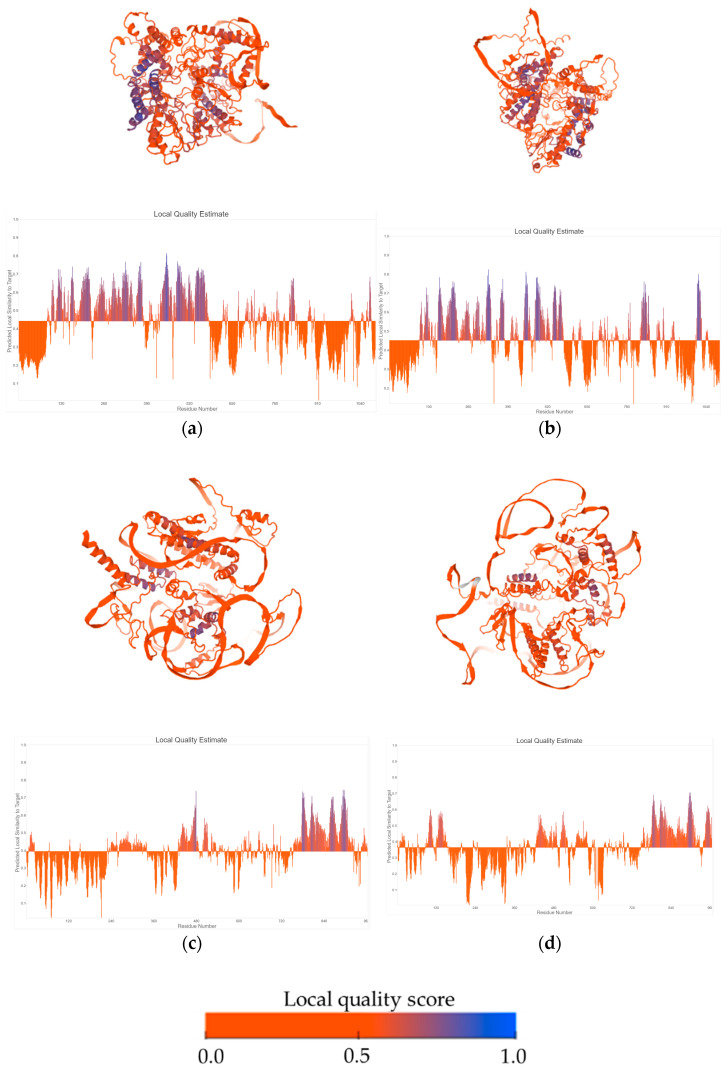
Predicted tertiary structures and local quality estimates of RdRp proteins of BNV1 (**a**) and BcNV1 (**b**); and hypothetical proteins of BNV1 (**c**) and BcBNV1 (**d**).

Tertiary structures of RdRp and hypothetical proteins were predicted with AlphaFold2 (Figure 3). RdRp proteins showed structural identity to polymerases of Human Picornavirus, among other ssRNA(+) viruses (Supplementary Figure S4). Further analysis of hypothetical protein encoded by BcNV1 indicated that its predicted tertiary structure showed structural homology with the envelope glycoprotein h of a herpesvirus and the capsid protein of white spot syndrome virus, with both viruses having a double-stranded DNA genome (Supplementary Figure S4). However, BNV1 ORF2-HP, did not show such a homology and was similar to aldolases (Supplementary Figure S4).

### 3.5. In Vivo Detection of Novel Mycoviruses

**Figure 4:**
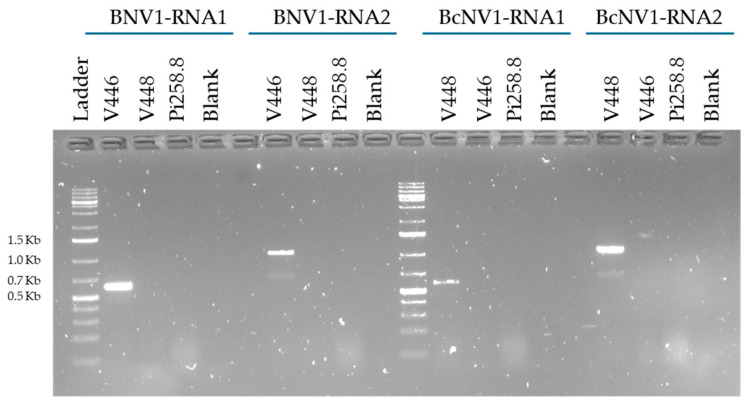
Detection of BNV1 and BcNV1 by RT-PCR amplification from infected samples V446 and V448. Size of several ladder bands is indicated.

To verify the presence of the novel mycoviruses BNV1 and BcNV1 in strains V446 and V448, respectively, RT and PCR amplification were performed with specific primers of each virus (Table 1; Figure 4). The amplified PCR products were Sanger sequenced to confirm the specific detection of both mycoviruses.

The selective amplification confirmed the presence of each mycoviral segment, RNA1 and RNA2 in BcNV1 or BNV1, in each corresponding infected strain, V448 or V446, respectively. The Sanger sequences obtained for BNV1 and BcNV1 were compared with the RNA-Seq sequences resulting in 100% of identity between them, confirming the presence of the novel mycoviruses identified in *B. cinerea* and *B. prunorum*.

### 3.6. Phylogenetic Relationships

**Figure 5:**
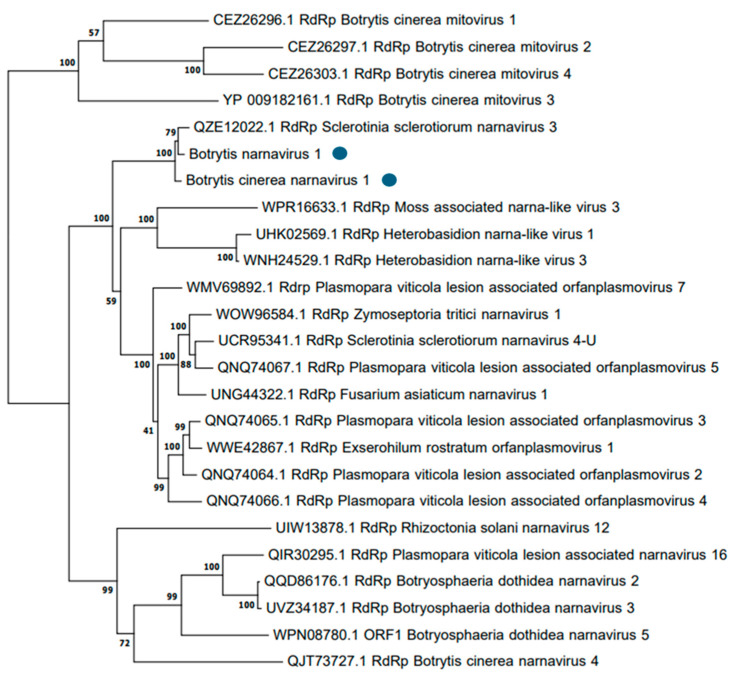
Phylogenetic tree computed by IQ-TREE stochastic algorithm to infer phylogenetic trees by maximum likelihood. Model of substitution: VT+I+G4. Consensus tree was constructed from 1000 bootstrap trees.

BNV1 and BcNV1 showed nucleotide and amino acid sequence identities according to the *BLAST* analysis conducted in the pipeline. A phylogenetic tree was constructed with the two newly identified mycoviruses, other narnaviruses previously found in *B. cinerea* and other bisegmented narnaviruses. Viruses classified in the genus *Mitovirus* were used as outgroups (Figure 5). Based on the phylogenetic tree computed with BcNV1 and BNV1 RdRp amino acid sequences, as expected, both mycoviruses were grouped in a separated group with strong bootstrap support (100% bootstrap value) with the bisegmented narnavirus SsNV-3, and in a different group than other narnavirus previously found in *B. cinerea* (Botrytis cinerea narnavirus 4, BcNV4).

## 4. Discussion

In this work, a reanalysis of the HTS data of three field isolates of two species of the genus *Botrytis* has been carried out. Isolate Pi258.8, from bell pepper, and isolate V448, obtained from grapevine, belong to the species *B. cinerea,* while isolate V446, also obtained from grapevine, belongs to the species *B. prunorum* [44]. In the present analysis of the three *Botrytis* field isolates, all the mycoviruses described in the first analysis of the data, performed by Donaire and Ayllón [32], have been identified. In addition, new mycoviruses not described in the aforementioned isolates were also found, including novel mycoviruses not previously detected infecting *Botrytis* species.

The efficacy of the bioinformatics pipeline used in this work had already been demonstrated in two previous studies. First, Chiapello et al. found 283 new viruses associated with *Plasmopara viticola* infection in grapevine crops [62], and, subsequently, Ruiz-Padilla et al. described 92 mycoviruses in different *Botrytis cinerea* field isolates obtained from grapevine plants [34]. In this work, we have compared the assembly performed by two assembly programs, *Trinity* [45] and *Spades* [46]. The results have shown that *Trinity* is indeed a more complete and more efficient assembler program than *Spades*. *Trinity* generated a larger number of assembled sequences (Table 2) as well as higher N50 values (Supplementary Table S2). This result aligns with the description of *Trinity* as the second best program for reconstructing ORFs [67] with the highest read alignment rate [68]. However, the computational time of *Trinity* is higher than that of *Spades*, which is generally low [69]. Regarding the assembly step, it has also been observed that, when reassembling with *CAP3*, the number of contigs assembled by *Trinity* that are joined together is greater than in the case of *Spades* (Table 2), due to the fact that by using different algorithms the same contigs are not obtained with each of them. Using both assembly methods, the same mycoviruses have been identified in the analyzed samples, so it can be stated that both assemblers have been effective.

In this work, the optimization of the bioinformatic pipeline as well as the update of the viral databases has led to the identification of several mycoviruses (BcAV1-Pi258.8, BcAV1-V448, PvaOLV80-V446, BNV1 of V446, BcNV1 of V448), not previously detected in these samples. BcAV1-Pi258.8 and -V448 are two isolates of BcAV1, which was first described in 2021 in *B. cinerea* isolates obtained from grapevine from Spain and Italy, and was the first alpha-like mycovirus identified that infected this fungus [34]. PvaOLV80-V446 should be classified as a mycovirus of the species *Deltascleroulivirus betaplasmoparae*. This species was first described in grapevines affected by infection with the plant pathogenic oomycete *P. viticola* [62]. It is a viral species that has not been found so far in *Botrytis* species, but since both are grapevine pathogens, it is tempting to speculate that both could have coinfected the same grapevine plant and that a horizontal transfer of this mycovirus could have occurred between *P. viticola* and *B. cinerea*. The horizontal transfer of mycoviruses between plant pathogenic fungus, such as *Botrytis* and *Sclerotinia* [34,70], has been documented and is considered a key aspect of RNA virus evolution [71]. It is also possible that the actual host of PvaOLV80 was *B. cinerea* instead of *P. viticola*, since Chiapello et al. [62] stated that this was a virus associated with the oomycete. In this mycovirome reanalysis, two novel narnaviruses, BNV1 of V446 and BcNV1 of V448, have been described and detected for the first time in *Botrytis* spp., increasing the large list of mycoviruses identified infecting this fungal genus, and suggesting that could be more associated mycoviruses to be discovered. Both are mycoviruses with bisegmented genomes, in which segment 1 codes for a RdRp and segment 2 for an HP. Although HP function is unknown, BcNV1 HP shows structural homology with capsid proteins of dsRNA viruses. Jia et al. [63] obtained similar results and speculated that the genome of SsNV-3 may not be naked, but encapsulated in virions. However, further analysis should be performed to determine the role of this HP in the mycoviral life cycle of BcNV1 and BNV1. Previously, narnaviruses with monosegmented genomes such as Botrytis cinerea narnavirus 1, or with multisegmented genomes, such as Botrytis cinerea binarnavirus 2, referred to as splipalmiviruses by some authors [72], have been found to be associated with *B. cinerea* [34]. However, splipalmiviruses are formed by two or more segments, in which RNA1 and RNA2 contain conserved motifs of the RdRp [11,13,34,73] and can also contain additional segments which code for hypothetical proteins with unknown functions [63]. Sequence identity analyses indicate that, evolutionarily, both novel *Botrytis* mycoviruses are closer to other different bisegmented narnaviruses rather than splipalmiviruses, such as those from *S. sclerotiorum* [63], *Heterobasidion* spp. [74] or *Fusarium asiaticum* [75]. They showed nucleotide and amino acid sequence identity among them, rather than to the other narnaviruses previously found in *B. cinerea*. These data were confirmed by the grouping of these mycoviruses in an independent clade in the phylogenetic analysis. Then, these novel mycoviruses are enlarging the list of new bisegmented narna-like viruses infecting *Botrytis* spp., and might be taxonomically classified in the family *Narnaviridae*.

On the other hand, an analysis of the mycovirus targets of the fungal gene silencing machinery has also been performed. It has been confirmed that all mycoviruses identified in our isolates, to a greater or lesser extent, are targeted by the *Botrytis* RNA-silencing machinery. This has been verified by mapping the sRNAs to the genomes of the mycoviruses. Most mycoviruses’ sRNAs have a size of 21 nts, except for SsNsV-L-V446, which comprises a higher amount of 22 nt vsRNAs, as has been found in the case of other dsRNA mycoviruses infecting *S. sclerotiorum* [76]. According to the results obtained, it has been observed that vsRNA abundances and viral RNA accumulation levels did not always correlate among all mycoviruses. Similar results were observed in dsRNAs mycoviruses infecting *Rosellinia necatrix* [77]. In general, it has been perceived that mycoviruses with shorter genomes, such as mitoviruses, produce more vsRNAs than mycoviruses with longer genomes, such as BVF, SsNsV-L, or BcAV1. Mitoviruses replicate in the mitochondria, so these results indicate that the fungal gene-silencing machinery is also operative within the mitochondria, as is the case in animals [78,79,80]. RNA-silencing machinery targeting mitoviruses was also previously described in other fungus besides *Botrytis* such as *Fusarium circinatum* [81]. There are various potential explanations for the divergent amounts of vsRNAs among mycoviruses, which may also shed light on whether a correlation exists between vsRNA levels and mycoviral accumulation. The biogenesis of vsRNAs depends on the formation of viral dsRNAs that are the substrate of the fungal dicer-like proteins (DCL) to generate vsRNAs of different sizes. Then, it is possible that mitoviruses replicate more actively, generating more dsRNAs than other mycoviruses, as has been previously observed in *B. cinerea* [29]; also, regions of complex secondary structures could encounter more dsRNAs in these small viral genomes [82]. The activity of fungal RNA-dependent RNA polymerases may also encounter more viral dsRNAs to generate secondary vsRNAs [83]. The activity of mycoviral suppressors of gene silencing (VSRs) proteins affecting the biogenesis or stability of vsRNAs may also explain these differences [84]. BVF produces small amounts of vsRNAs, indicating a low level of DCL targeting, which may be due to CP protecting the mycovirus genome from host gene-silencing proteins as has been suggested for other mycoviruses [85] or due to the presence of an associated VSR protein. With respect to the novel mycoviruses found in *Botrytis* isolates, BcAV1-Pi258.8 is the one with the highest expression level and with the lowest vsRNA accumulation; a possible explanation for this could be that this mycovirus also contains a VSR protein that prevents its effective degradation by the RNA-silencing machinery. In both cases, BVF and BcAV1, additional studies should be performed to confirm this hypothesis. However, PavOLV80-V446 is shown to be commensurate with its expression level. Narnaviruses are ssRNA(+) that replicate in the cytosol [86], and are possible targets of the fungal RNA-silencing machinery. Indeed, a narnavirus detected in *P. castaneae* was targeted by the oomycete-silencing machinery [87], as well as a narna-like virus in the bush *Viburnum odoratissimum*; sRNA-Seq data suggested that the virus actively replicated within the host plant and interacted with its antiviral pathways [88]. In this work, vsRNAs derived from both novel bisegmented narnaviruses were found. Interestingly, the new narnaviruses present differences that should be highlighted. BcNV1 accumulated at lower levels than BNV1, and while in BcNV1 both genomic segments are targeted by the silencing machinery of the fungal host at the same level, for BNV1, segment 2 is processed by fungal DCL more effectively. This could be explained by the fact that, in the case of BcNV1, both segments present similar levels of expression, and, on the contrary, in BNV1, the second segment is more highly expressed than the first one. Finally, it was also observed that BcNV1 is apparently more silenced than BNV1, and a possible hypothesis for this could be that the host’s (*B. cinerea* or *B. prunorum*) silencing machinery is differentially targeting these types of viruses. Since GaNV-1 was misclassified as a narnavirus, and subsequently renamed Botrytis cinerea mitovirus 9 [34], it is assumed according the results obtained in this work that, for the first time, narnaviruses have been described as targets of the *Botrytis* spp.-gene silencing machinery. Additionally, studies should be performed to dissect the complete gene-silencing mechanism in the antiviral defense of *Botrytis* species.

## Figures and Tables

**Table 1:** Primers for detection of Botrytis cinerea narnavirus 1, Botrytis narnavirus 1 and Plasmopara viticola lesion-associated ourmia-like virus 80.

Primer	Sequence (5’-3’)	Virus-Segment Targeted	Position of Primers
BNV1 F1	AGAAGGGTATTTGGATAGGTTCGC	BNV1-RNA1	1006–1029
BNV1 R1	TCTTGGGAATACCAATCGCCAGAC	BNV1-RNA1	1591–1614
		BcNV1-RNA1	1556–1579
BcNV1 F2	AAGGTCATCCCTAAACAGGAGAT	BNV1-RNA2	1280–1302
		BcNV1-RNA2	1286–1308
BNV1 R2	GATTCTAAAATTTCCTTTGGGATAGCTT	BNV1-RNA2	2325–2352
BcNV1 F1	ACGTTTTAAACCTAAGTTTACGTCCTC	BcNV1-RNA1	992–1018
BcNV1 R2	TCTAAAACCTCATGGGATATTACCC	BcNV1-RNA2	2338–2362
PvaOLV80 F	CCGGTTCCTTCGTTTCCGTTGACTTC	RNA	942–967
PvaOLV80 R	CCCATCATCTGTCCCATCGAAAGC	RNA	1174–1197

Expected lengths for detection of each segment were: BNV1 RNA1, 609 bp; BNV1 RNA2, 1073 bp; BcNV1 RNA1, 588 bp; BcNV1 RNA2, 1077 bp; PvaOLV80, 256 bp.

**Table 2:** Comparison of the performances of *Trinity* and *Spades*. Number of contigs (**#**) assembled by *Trinity* and *Spades*; the number of the contigs with significant. * *BLAST*x hits on viral database and the number of viral contigs after reassembly with *CAP3*.

Isolates	*Trinity*			*Spades*		
	# Contigs	# Hits	# Hits *CAP3*	# Contigs	# Hits	# Hits *CAP3*
Pi258.8	14.178	3.278	2.956	10.967	2.574	2.524
V446	15.209	3.612	3.293	11.671	2.751	2.688
V448	19.205	4.815	4.314	13.770	3.440	3.339

* e-value = 0.00001.

**Table 3:** Sequences similar to mycoviruses identified in *Botrytis* isolates Pi258.8, V446 and V448. The family and genus to which each mycovirus belongs, its genome type and its Accession number (acc. No.) for nucleotide and protein sequences are indicated in the table.

Field Isolate	Mycovirus *	Family	Genus	Genome	Acc. No.
**Pi258.8**	BcMV-1	*Mitoviridae*	*Duamitovirus*	(+)ssRNA	LN827940/CEZ26296
	BcMV-2	*Mitoviridae*	*Unuamitovirus*	(+)ssRNA	LN827941/CEZ26297
	BcMV-3	*Mitoviridae*	*Duamitovirus*	(+)ssRNA	LN827942/CEZ26298
	GaNV-1	*Mitoviridae*	*Mitovirus*	(+)ssRNA	LN827943/CEZ26299
	**BcAV1**	*Togaviridae*	*Alphavirus*	(+)ssRNA	MN625250/QJT73733
**V446**	BOLV	*Botourmiaviridae*	*Botoulivirus*	(+)ssRNA	LN827955/CEZ26310
	SsNsV-L	Unclassified	Unclassified	dsRNA	LN827951/CEZ26307
	**PvaOLV80**	*Botourmiaviridae*	*Deltascleroulivirus*	(+)ssRNA	MN532667/QGY72610
	**SsNV-3 (RNA1)**	*Narnaviridae*	*Narnavirus*	(+)ssRNA	MW442873/QZE12022
	**SsNV-3 (RNA2)**	*Narnaviridae*	*Narnavirus*	(+)ssRNA	MW442874/QZE12023
**V448**	BcMV-1	*Mitoviridae*	*Duamitovirus*	(+)ssRNA	LN827944/CEZ26300
	BcMV-2	*Mitoviridae*	*Unuamitovirus*	(+)ssRNA	LN827945/CEZ26301
	BcMV-3	*Mitoviridae*	*Duamitovirus*	(+)ssRNA	LN827946/CEZ26302
	BcMV-4	*Mitoviridae*	*Unuamitovirus*	(+)ssRNA	LN827947/CEZ26303
	GaNV-1	*Mitoviridae*	*Mitovirus*	(+)ssRNA	LN827948/CEZ26304
	SsMV-3	*Mitoviridae*	*Duamitovirus*	(+)ssRNA	LN827949/CEZ26305
	SsNsV-L	Unclassified	Unclassified	dsRNA	LN827952/CEZ26308
	BVF	*Gammaflexiviridae*	*Mycoflexivirus*	(+)ssRNA	LN827954/CEZ26309
	BcNSRV-1	Unclassified	Unclassified	(−)ssRNA	LN827956/CEZ26311
	**BcAV1**	*Togaviridae*	*Alphavirus*	(+)ssRNA	MN625250/QJT73733
	**SsNV-3 (RNA1)**	*Narnaviridae*	*Narnavirus*	(+)ssRNA	MW442873/QZE12022
	**SsNV-3 (RNA2)**	*Narnaviridae*	*Narnavirus*	(+)ssRNA	MW442874/QZE12023

* Botrytis cinerea mitovirus 1 (BcMV-1); Botrytis cinerea mitovirus 2 (BcMV-2); Botrytis cinerea mitovirus 3 (BcMV-3); Grapevine-associated narnavirus 1 (GaNV-1); Botrytis cinerea alpha-like virus 1 (BcAV1); Botrytis ourmia-like virus (BOLV); Sclerotinia sclerotiorum dsRNA mycovirus-L (SsNsV-L); Plasmopara viticola lesion associated ourmia-like virus 80 (PvaOLV80); Sclerotinia sclerotiorum narnavirus 3 (SsNV-3); Sclerotinia sclerotiorum narnavirus 3 (SsNV-3) (mycovirus sequences found in this work with identity to SsNV-3 have been named Botrytis cinerea narnavirus 1 (BcNV1) in the V448 isolate and Botrytis narnavirus 1 (BNV1) in the V446 isolate, respectively); Botrytis cinerea mitovirus 4 (BcMV-4); Sclerotinia sclerotiorum mitovirus-3 (SsMV-3); Botrytis virus F (BVF); Botrytis cinerea negative-stranded RNA virus 1 (BcNSRV-1). Mycoviruses that were not previously identified in these samples are labeled in bold.

**Table 4:** Summary of the analysis of novel mycoviral sequences in the isolates. The following information is indicated for each mycovirus sequence: contig length (nt), contig coverage on reference genome, contig nt and aa identity with the mycoviral reference genome, and whether the CDS is complete.

Field Isolate *	Mycoviral Reference Genome	Mycoviral Reference Genome Length (nt)	Mycoviral Reference Genome CDS (aa)	Mycoviral Reference Genome CDS Domain	Contig Length (nt)	Contig Coverage on Reference Genome (aa)	Contig nt Identity (%)	Contig aa Identity (%)	Complete CDS
Pi258.8	BcAV1	8008	1975	RdRp	8045	100	96	99	Yes
V446	PavOLV80	3000	737	RdRp	3146	100	96	98	Yes
	SsNV-3 (RNA1)	3453	1087	RdRp	3447	100	77	83	Yes
	SsNV-3 (RNA2)	3100	957	HP	3108	100	58.5	61	Yes
V448	BcAV1	8008	1975	RdRp	6177	76.1	95	99	No
	SsNV-3 (RNA1)	3453	1087	RdRp	3437	100	79.5	80	Yes
	SsNV-3 (RNA2)	3100	957	HP	3120	100	56.8	58	Yes

* Mycoviral reference genome length (nt): length of the genome or individual segments in nucleotides. Mycoviral reference genome CDS (aa): length of proteins encoded by the genome or individual segments in amino acids. Mycoviral reference genome CDS domain: RdRp (RNA dependent RNA polymerase) or HP (Hypothetical protein). Contig length (nt): length of assembled mycoviral contigs in nucleotides. Contig coverage on reference genome (aa): percentage of the alignment between mycoviral contigs and mycoviral genome/segments in amino acids. Conting nt identity (%): percentage of identity at nucleotide level. Conting aa identity (%): percentage of identity at amino acid level. Complete CDS: indicates if the mycoviral contigs have been assembled to form full mycoviral reference CDS sequences.

## Data Availability

RNAseq raw reads are available in the Sequence Read Archive (SRA) at NCBI: BioProject PRJNA325479 (https://www.ncbi.nlm.nih.gov/bioproject/PRJNA325479, accessed on 28 July 2024). Mycoviral sequences are available in the GenBank^®®^ database (https://www.ncbi.nlm.nih.gov/genbank/, accessed on 28 July 2024). Total number of ontigs resulted from the assembly were not uploaded to the databases.

## Supplementary Materials

The following supporting information can be downloaded at: https://www.mdpi.com/article/10.3390/v16101640/s1, Figure S1: Mapping data of mycoviruses; Figure S2: Genome schemes and RT-PCR results; Figure S3: Distribution of sRNAs; Figure S4: Predicted tertiary structures of RdRp and HP proteins of BNV1 and BcNV1; Table S1: Range of contigs length; Table S2: N50 values.
