# Supplementary material for: Reexamining the Mycovirome of Botrytis spp
Source: Viruses. 2024 Oct 21;16(10):1640. doi: 10.3390/v16101640 (PMC11512270; doi:10.3390/v16101640)
Supplement: Supplementary file 1 [file viruses-16-01640-s001.zip › Supplementary Figure S1 Muñoz-Suárez et al. 2024.pdf]

Supplementary Figure S1. Mapping data of mycoviruses. Schemes show full-genomes and reads alignments along the genome.

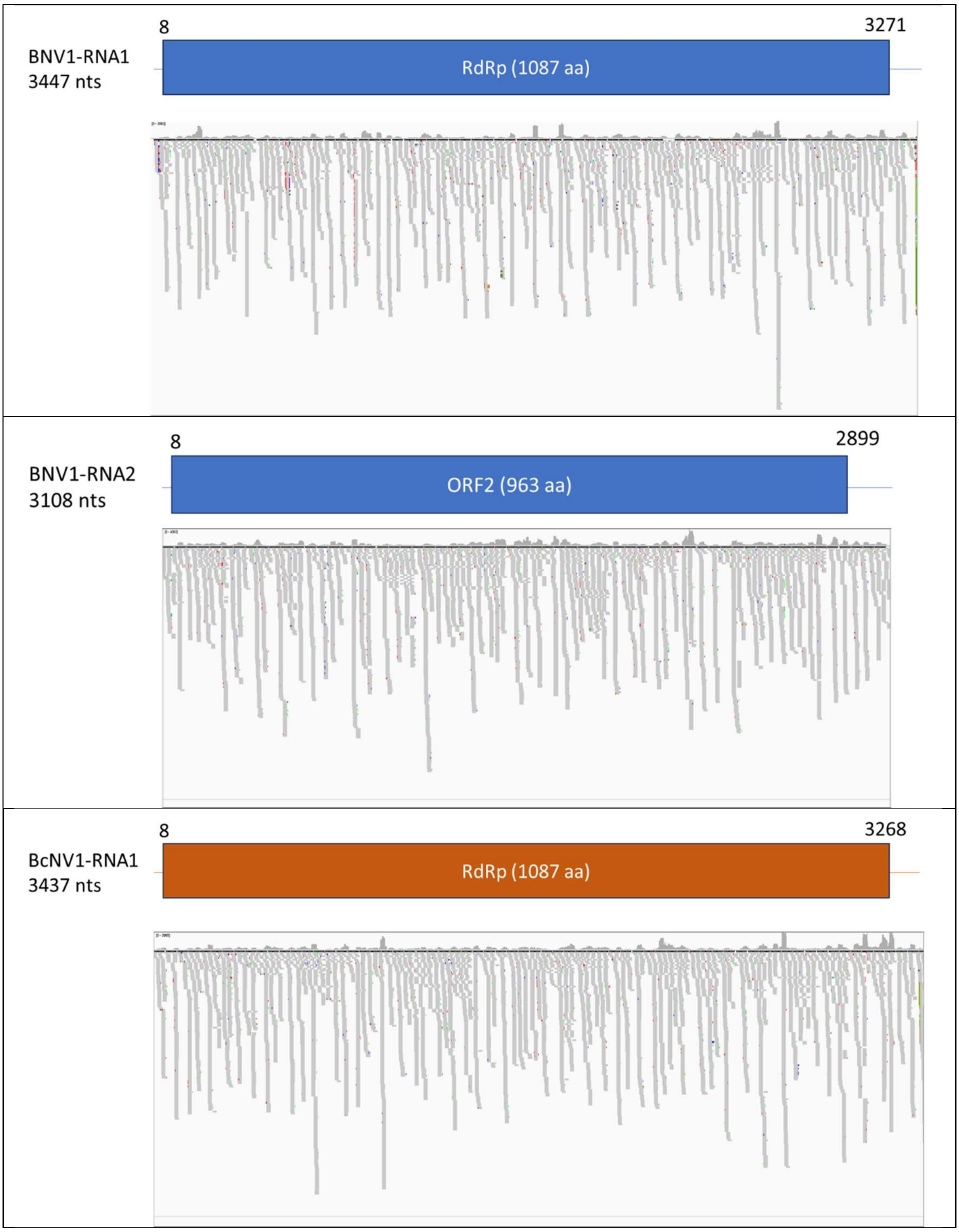

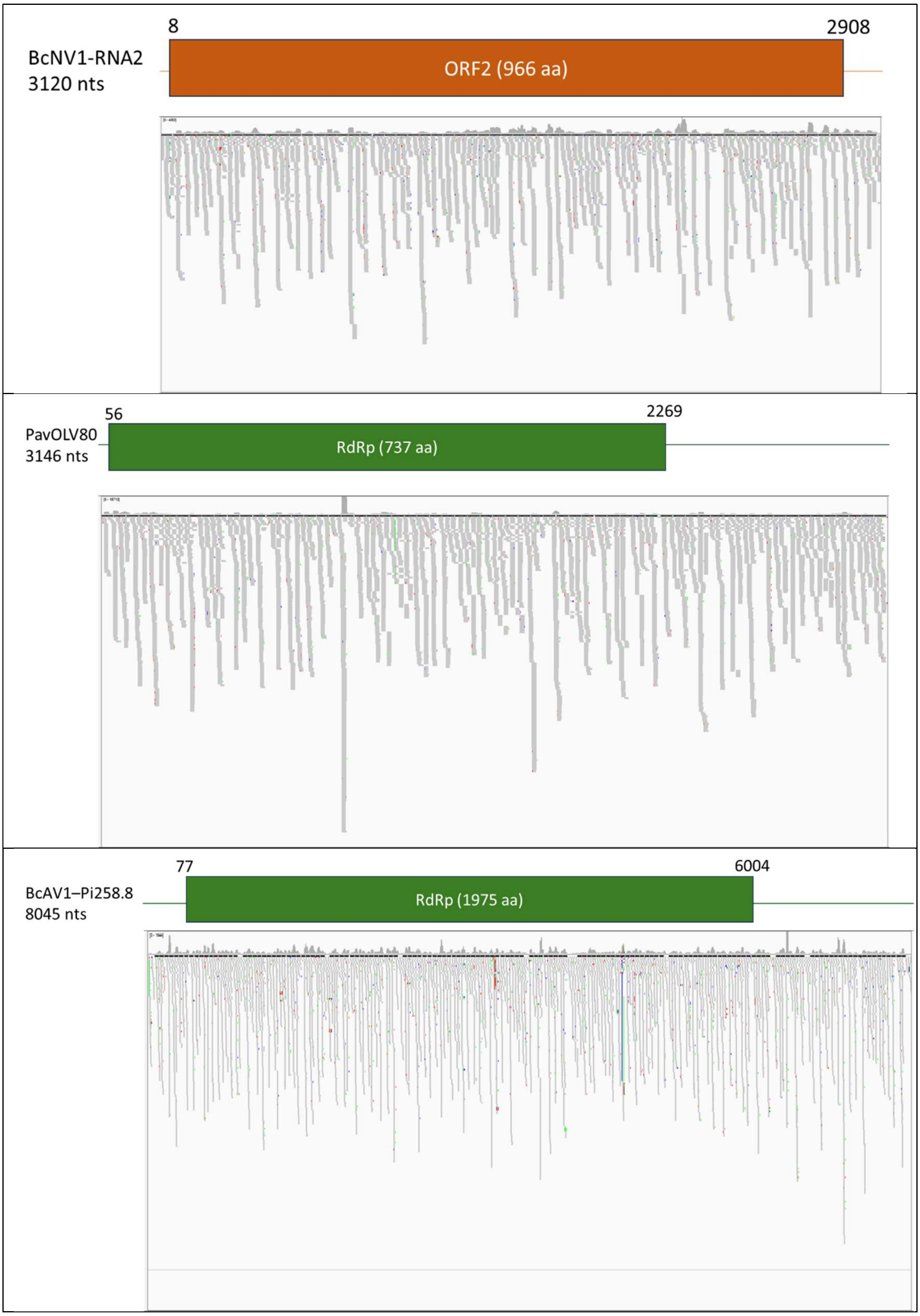

BcNV1-RNA2
3120 nts
8
2908
ORF2 (966 aa)
PavOLV80
3146 nts
56
2269
RdRp (737 aa)
BcAV1–Pi258.8
8045 nts
77
6004
RdRp (1975 aa)

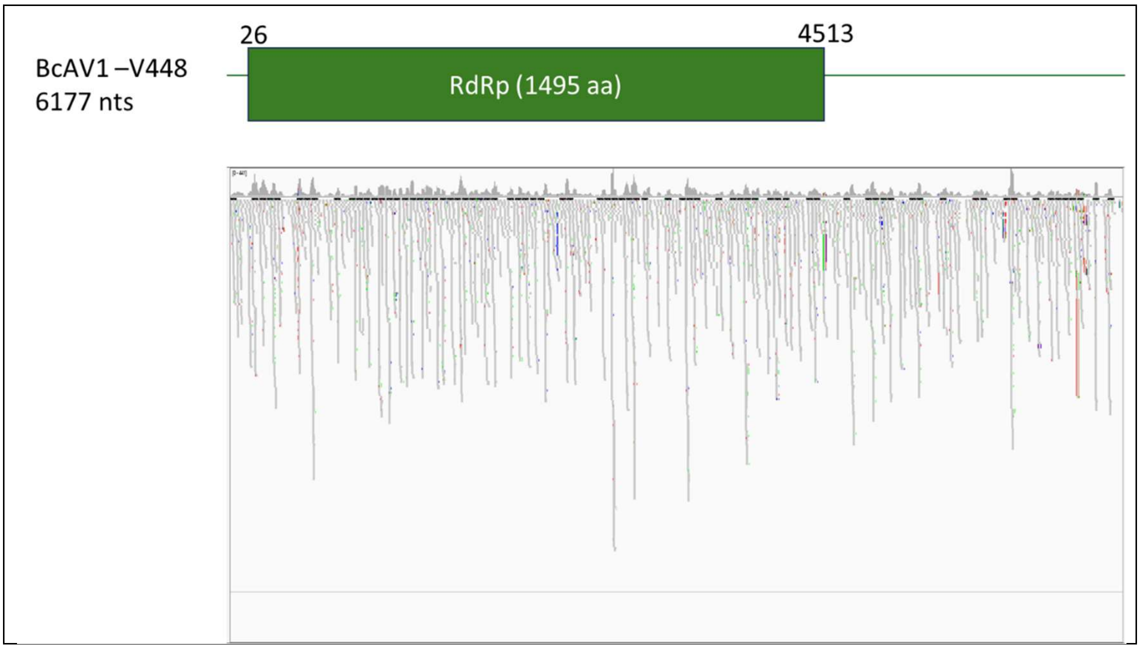

BcAV1 –V448
6177 nts
26
4513
RdRp (1495 aa)
