# Supplementary material for: Reexamining the Mycovirome of Botrytis spp
Source: Viruses. 2024 Oct 21;16(10):1640. doi: 10.3390/v16101640 (PMC11512270; doi:10.3390/v16101640)
Supplement: Supplementary file 1 [file viruses-16-01640-s001.zip › Supplementary Figure S2 Muñoz-Suárez et al. 2024 v2.pptx]

## Slide 1
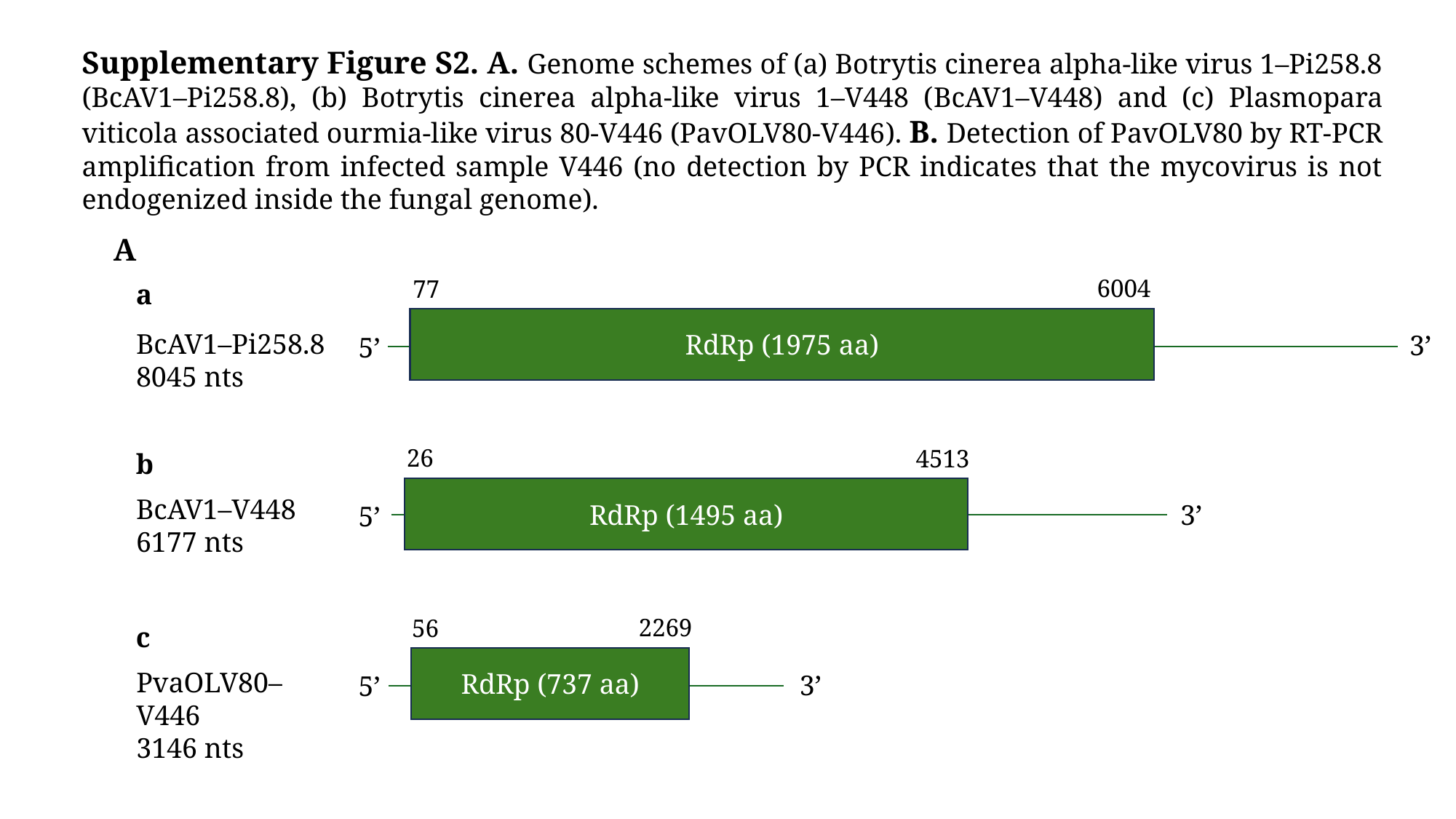

Supplementary Figure S2. A. Genome schemes of (a) Botrytis cinerea alpha-like virus 1–Pi258.8 (BcAV1–Pi258.8), (b) Botrytis cinerea alpha-like virus 1–V448 (BcAV1–V448) and (c) Plasmopara viticola associated ourmia-like virus 80-V446 (PavOLV80-V446). B. Detection of PavOLV80 by RT-PCR amplification from infected sample V446 (no detection by PCR indicates that the mycovirus is not endogenized inside the fungal genome).
A
6004
77
RdRp (1975 aa)
3’
5’
a
BcAV1–Pi258.8
8045 nts
26
4513
RdRp (1495 aa)
3’
5’
b
BcAV1–V448
6177 nts
2269
56
RdRp (737 aa)
3’
5’
c
PvaOLV80–V446
3146 nts

## Slide 2
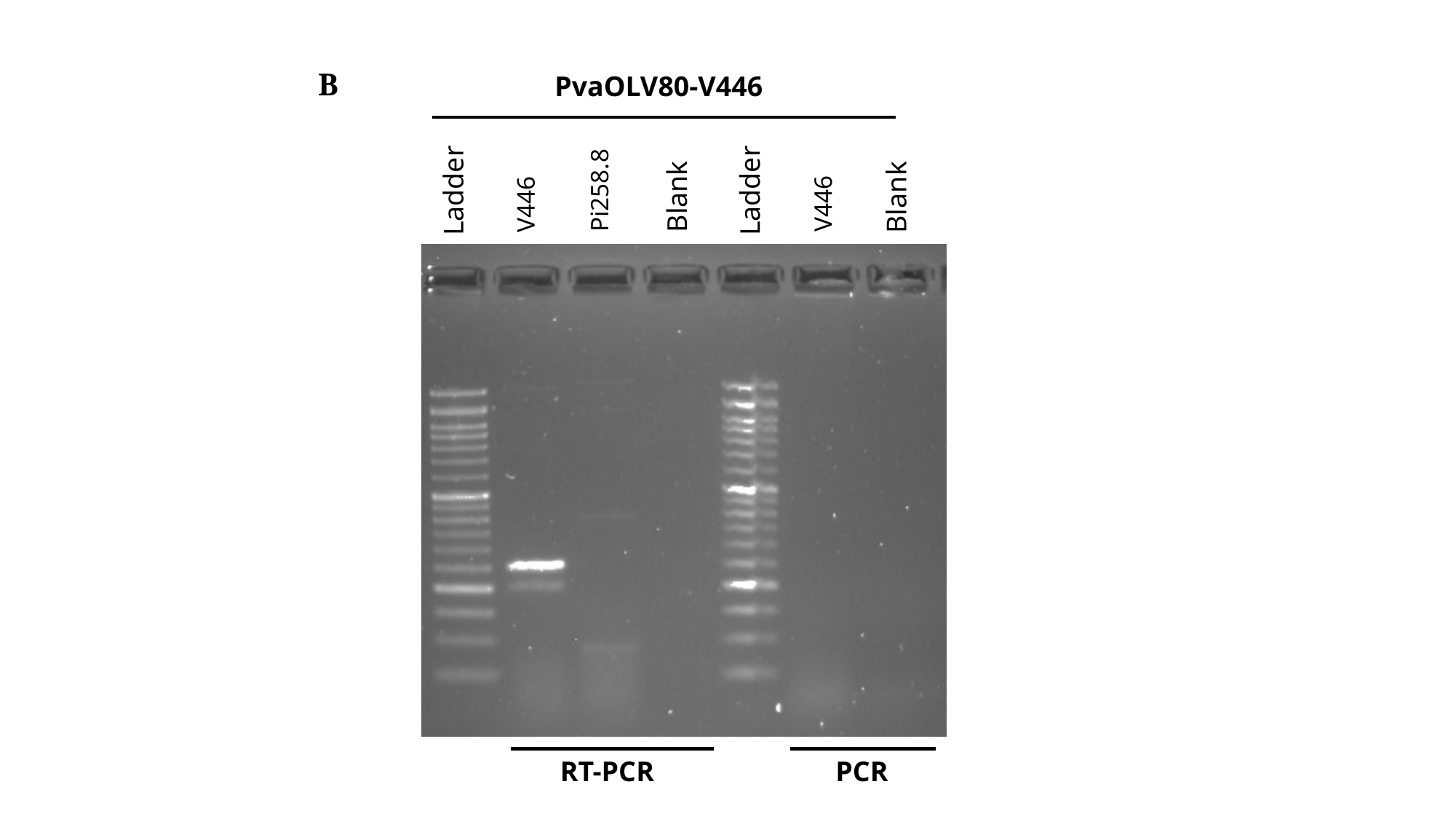

B
PvaOLV80-V446
Pi258.8
Ladder
Ladder
Blank
Blank
V446
V446
RT-PCR
PCR
