# Supplementary material for: Reexamining the Mycovirome of Botrytis spp
Source: Viruses. 2024 Oct 21;16(10):1640. doi: 10.3390/v16101640 (PMC11512270; doi:10.3390/v16101640)
Supplement: Supplementary file 1 [file viruses-16-01640-s001.zip › Supplementary Figure S3 Muñoz-Suárez et al. 2024.pptx]

## Slide 1
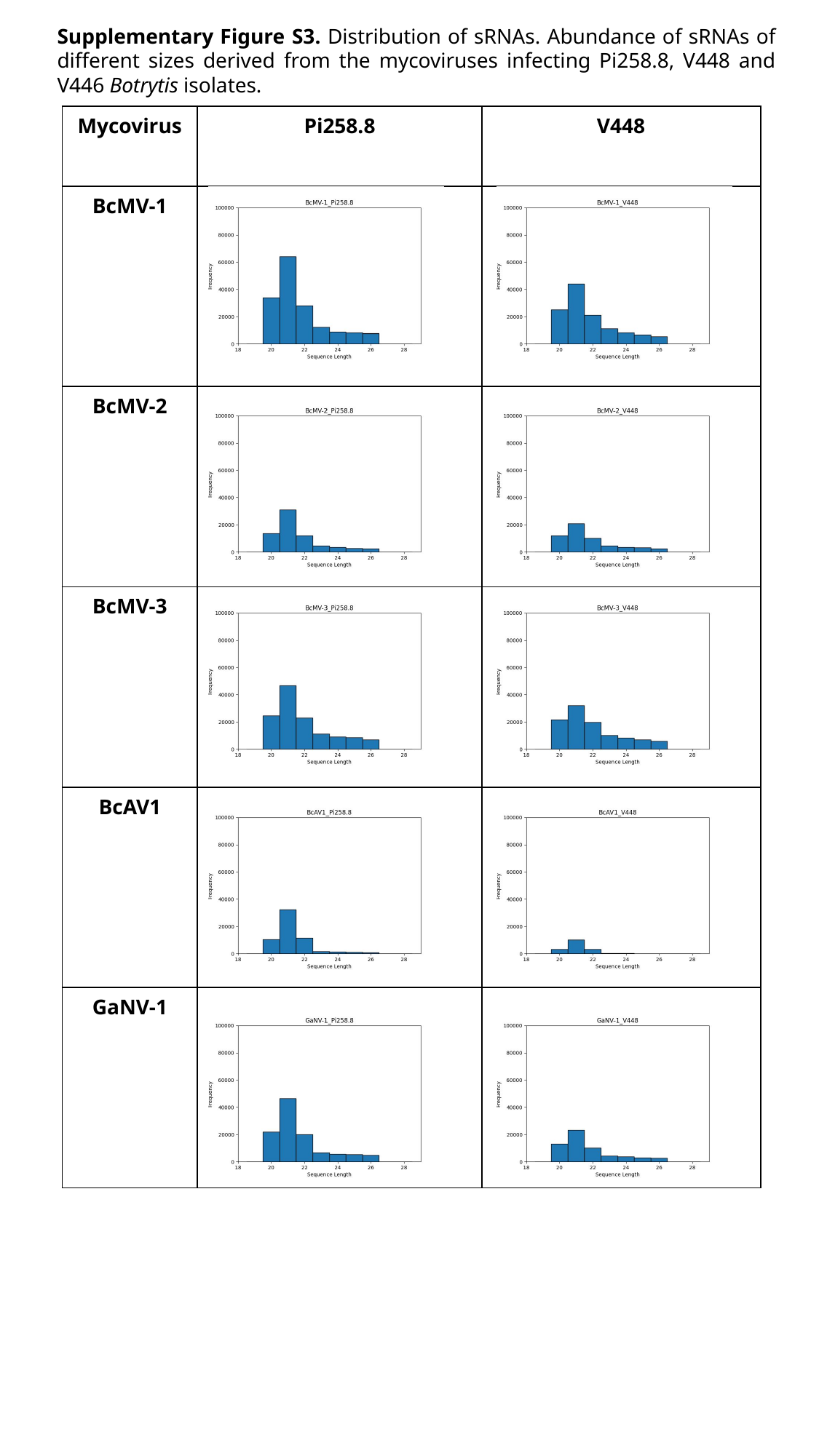

Supplementary Figure S3. Distribution of sRNAs. Abundance of sRNAs of different sizes derived from the mycoviruses infecting Pi258.8, V448 and V446 Botrytis isolates.
| Mycovirus | Pi258.8 | V448 |
| --- | --- | --- |
| BcMV-1 | | |
| BcMV-2 | | |
| BcMV-3 | | |
| BcAV1 | | |
| GaNV-1 | | |

## Slide 2
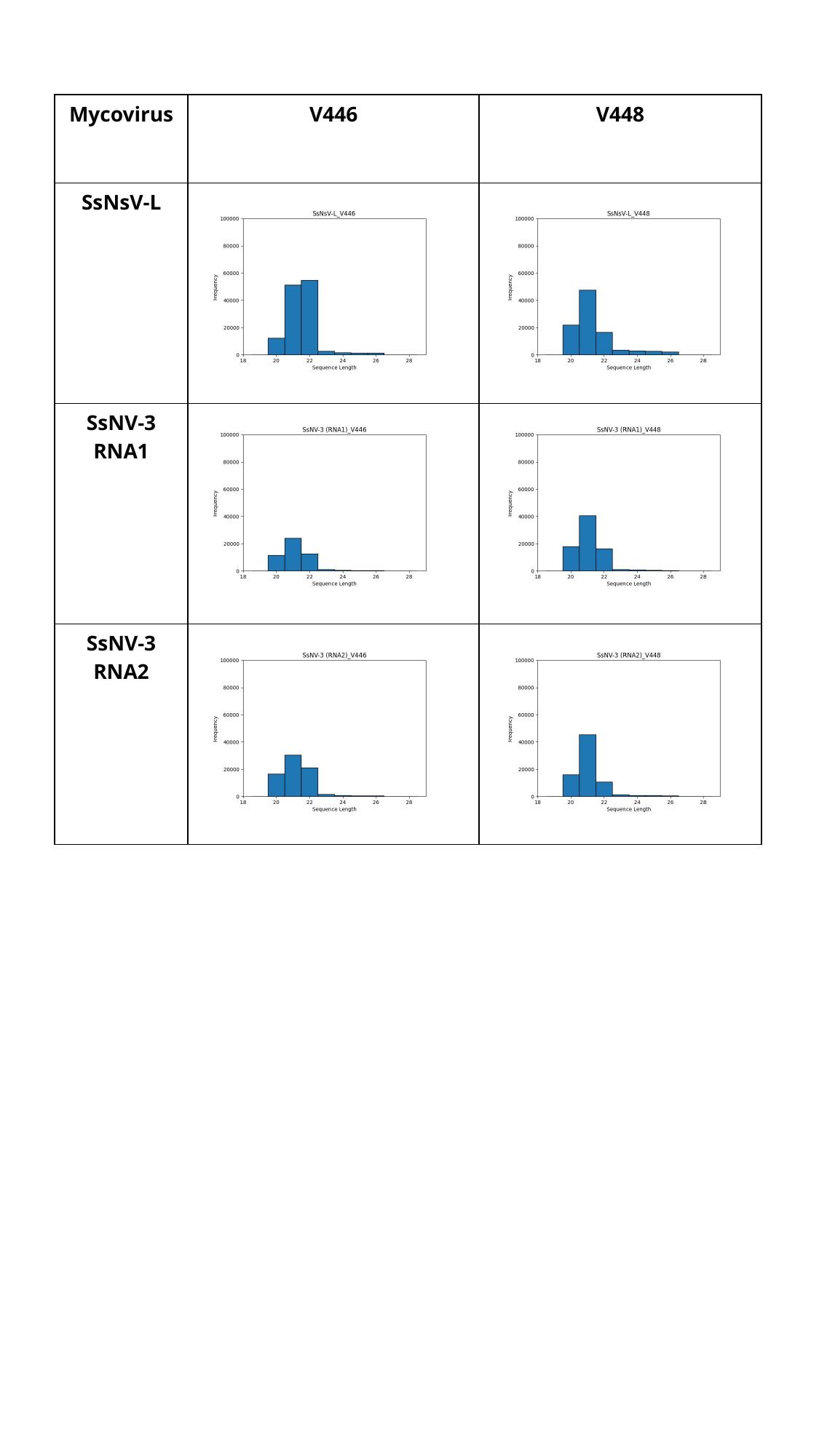

| Mycovirus | V446 | V448 |
| --- | --- | --- |
| SsNsV-L | | |
| SsNV-3 RNA1 | | |
| SsNV-3 RNA2 | | |

## Slide 3
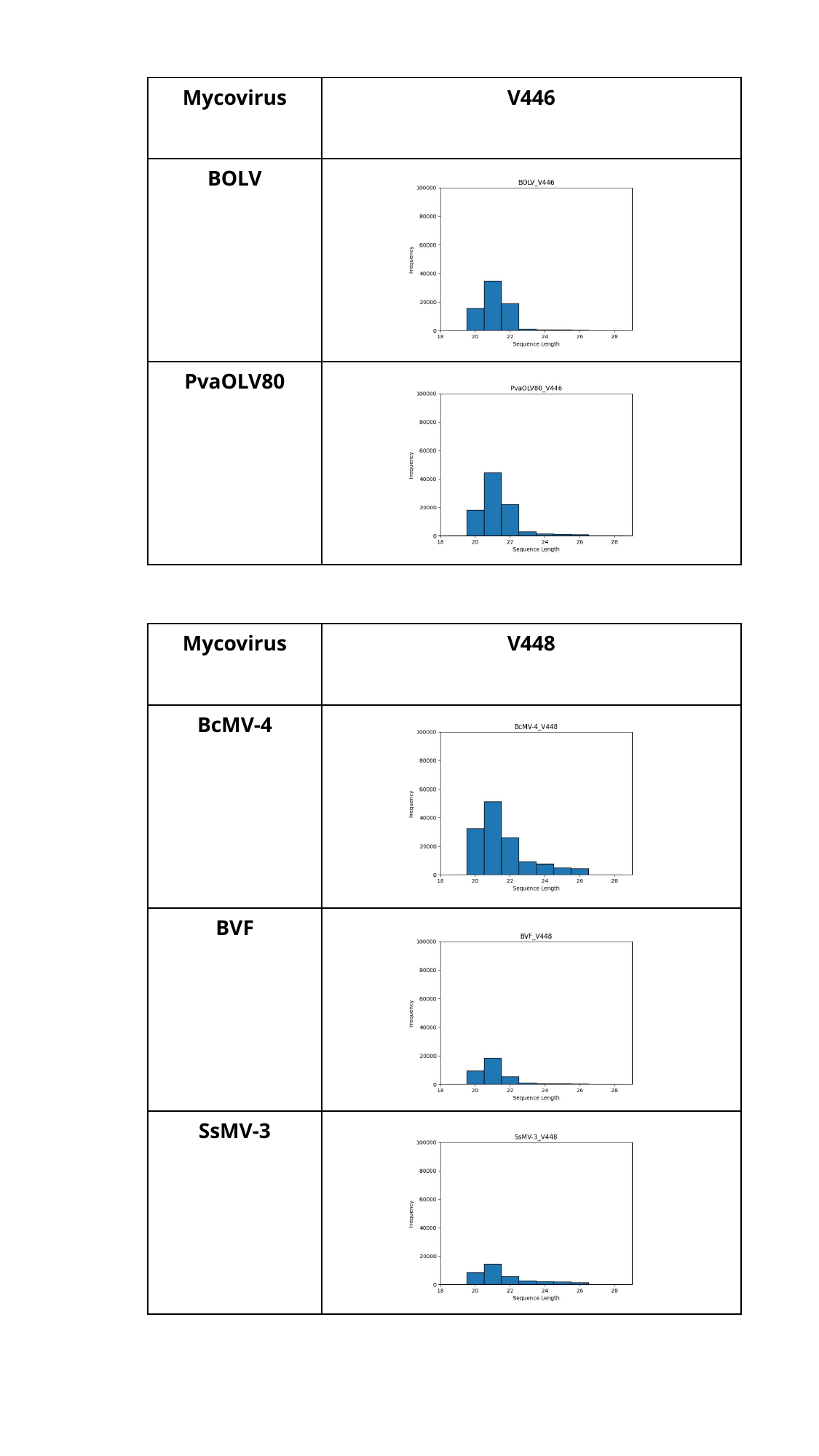

| Mycovirus | V446 |
| --- | --- |
| BOLV | |
| PvaOLV80 | |
| Mycovirus | V448 |
| --- | --- |
| BcMV-4 | |
| BVF | |
| SsMV-3 | |
