# Supplementary material for: Reexamining the Mycovirome of Botrytis spp
Source: Viruses. 2024 Oct 21;16(10):1640. doi: 10.3390/v16101640 (PMC11512270; doi:10.3390/v16101640)
Supplement: Supplementary file 1 [file viruses-16-01640-s001.zip › Supplementary Figure S4 Muñoz-Suárez et al. 2024.pptx]

## Slide 1
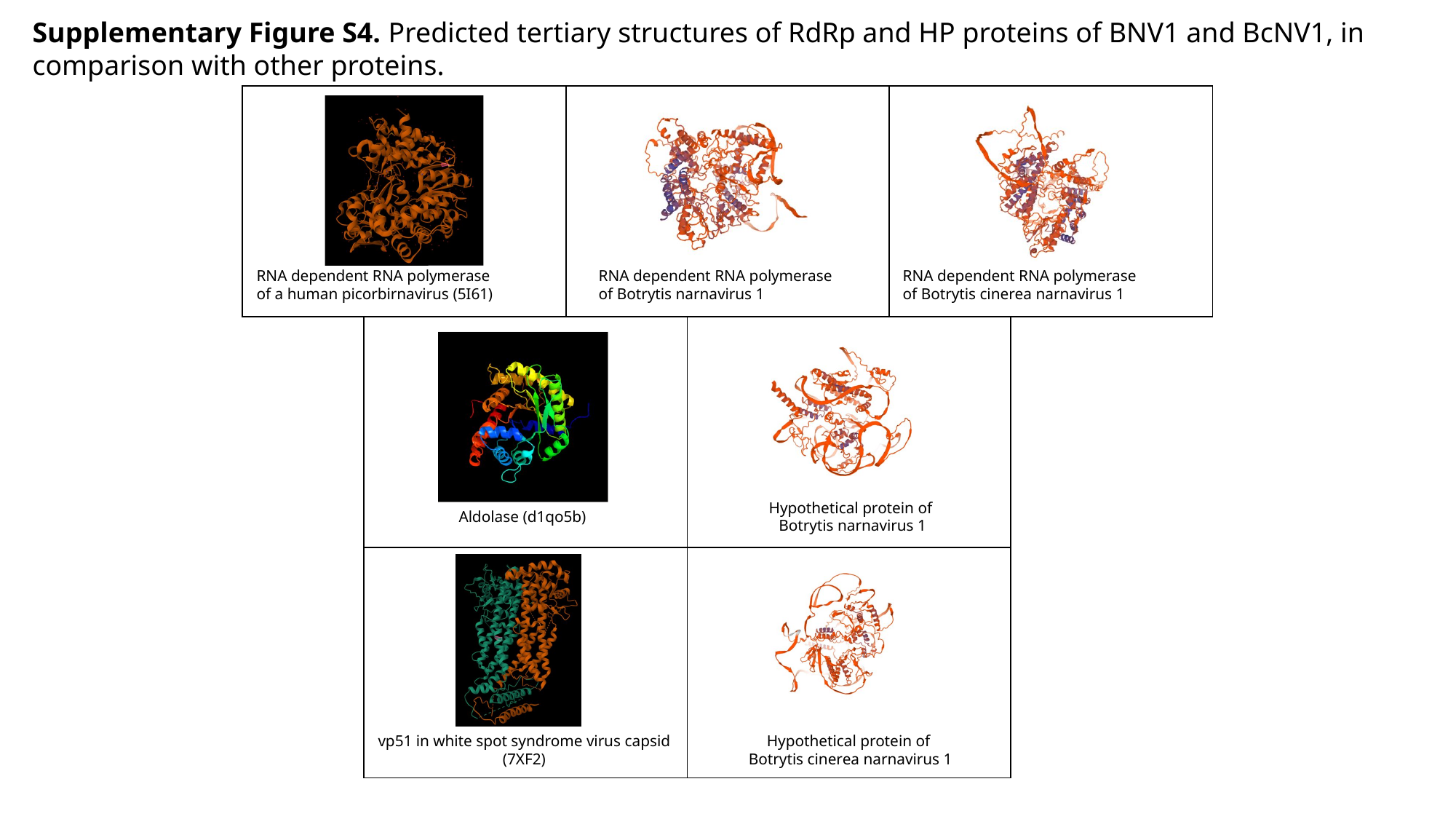

Supplementary Figure S4. Predicted tertiary structures of RdRp and HP proteins of BNV1 and BcNV1, in comparison with other proteins.
| | | |
| --- | --- | --- |
RNA dependent RNA polymerase
of a human picorbirnavirus (5I61)
RNA dependent RNA polymerase
of Botrytis narnavirus 1
RNA dependent RNA polymerase
of Botrytis cinerea narnavirus 1
| | |
| --- | --- |
| | |
Hypothetical protein of
Botrytis narnavirus 1
Aldolase (d1qo5b)
vp51 in white spot syndrome virus capsid (7XF2)
Hypothetical protein of
Botrytis cinerea narnavirus 1
