# Supplementary material for: Reexamining the Mycovirome of Botrytis spp
Source: Viruses. 2024 Oct 21;16(10):1640. doi: 10.3390/v16101640 (PMC11512270; doi:10.3390/v16101640)
Supplement: Supplementary file 1 [file viruses-16-01640-s001.zip › Supplementary Tables Muñoz-Suárez et al. 2024 v2.pdf]

Supplementary Tables

Supplementary Table S1. Range of contigs length (minimum-maximum) of isolates Pi258.8, V446, V448.

Length value range	Spades contigs	Spades viral contigs before CAP3	Spades viral contigs after CAP3	Trinity contigs	Trinity viral contigs before CAP3	Trinity viral contigs after CAP3
V446	49-21541	162-13683	162-13683	201-21775	217-10795	217-10795
V448	49-15035	156-15035	156-15035	201-10795	209-10795	209-10795
Pi258.8	50-11311	167-11311	167-11311	201-10795	216-10795	216-10795

Supplementary Table S2. N50 values of assembled contigs of isolates Pi258.8, V446, V448.

N50	Spades contigs	Spades viral contigs before CAP3	Spades viral contigs after CAP3	Trinity contigs	Trinity viral contigs before CAP3	Trinity viral contigs after CAP3
V446	2185	2405	2405	2288	2793	2822
V448	2349	2614	2636	2348	2827	2825
Pi258.8	2067	2256	2270	2089	2419	2467
